# Cytomorphometric and Clinical Analysis of the Effects of Azithromycin and Platelet-Rich Fibrin on Wound Healing After Surgical Removal of an Impacted Mandibular Third Molar

**DOI:** 10.3390/jfb17060307

**Published:** 2026-06-21

**Authors:** Milan Spasić, Kosta Todorović, Nikola Živković, Milica Petrović, Simona Stojanović, Ana Todorović, Branislava Stojković, Sanja Jocić, Vladan Krunić, Milan Stoiljković

**Affiliations:** 1 Department of Oral Surgery, Dental Clinic, Faculty of Medicine, University of Niš, 18108 Niš, Serbia; milan.s.spasic@gmail.com (M.S.); kosta.todorovic@medfak.ni.ac.rs (K.T.); simona.stojanovic@medfak.ni.ac.rs (S.S.); sanjajocic1997@gmail.com (S.J.); krunicv@yahoo.com (V.K.); 2 Department of Pathology, Institute of Pathological Anatomy, Clinical Center, Faculty of Medicine, University of Niš, 18108 Niš, Serbia; nikola.zivkovic@medfak.ni.ac.rs; 3 Department of Oral Medicine and Periodontology, Dental Clinic, Faculty of Medicine, University of Niš, 18108 Niš, Serbia; 4 Department of Orthodontics, Dental Clinic, Faculty of Medicine, University of Niš, 18108 Niš, Serbia; ana.todorovic@medfak.ni.ac.rs; 5 Department of Preventive and Pediatric Dentistry, Dental Clinic, Faculty of Medicine, University of Niš, 18108 Niš, Serbia; branislava.stojkovic@medfak.ni.ac.rs; 6 Department of Pharmacology, Faculty of Medicine, University of Niš, 18108 Niš, Serbia; milan.stojiljkovic@medfak.ni.ac.rs; 7 Department of Comparative Medicine, Yale University School of Medicine, New Haven, CT 06510, USA

**Keywords:** wisdom tooth, biomaterials, cytomorphometric analysis, regenerative dentistry, Azithromycin, PRF

## Abstract

Impacted mandibular third molars present a common challenge in oral surgery, often associated with postoperative complications such as delayed healing and periodontal defects; therefore, optimizing adjunctive therapies is clinically important. In this study, we aimed to evaluate the efficacy of platelet-rich fibrin (PRF) and preoperative azithromycin in modulating inflammation and enhancing wound healing following surgical extraction of impacted mandibular third molars. In this prospective clinical study, healthy subjects aged 18–50 years were randomly assigned to three groups: a control group receiving standard postoperative amoxicillin therapy, a PRF group receiving PRF with standard therapy, and a PRF-plus-azithromycin group receiving PRF, standard therapy, and a single preoperative dose of azithromycin. Clinical parameters were assessed and cytomorphometric analysis was performed preoperatively and postoperatively. Clinical parameters generally improved over time in all groups (*p* < 0.001). Differences between groups were observed for interincisal distance, Landry Index, and pain scores, with a trend toward more favorable outcomes in the combined-therapy group. Cytomorphometric analysis revealed cellular alterations in the control group, relative stability in the PRF group, and intermediate changes in the combined-therapy group. Within the limitations of this study, the combination of PRF and preoperative azithromycin showed potential benefits in several postoperative outcomes. However, given the study design and sample characteristics, these findings should be considered preliminary and require confirmation in larger prospective studies before definitive clinical recommendations can be made.

## 1. Introduction

Tooth impaction represents a condition in which a tooth fails to erupt completely into its normal functional position in the dental arch due to a lack of space, abnormal position, or obstruction by surrounding hard or soft tissues [1]. Impaction may occur in association with hereditary syndromes, endocrine disorders, radiotherapy, malposition of tooth germs, supernumerary teeth, odontogenic tumors, or other conditions [2]. The prevalence of impacted teeth in the general population is estimated at 36.9%, with mandibular third molars being the most commonly affected, followed by maxillary third molars, maxillary canines, and other teeth [3].

The surgical extraction of impacted third molars plays a key role in maintaining both oral and general health and represents one of the most commonly performed procedures in oral surgery [4]. Impacted third molars pose a significant challenge in both treatment planning and surgical management. Adequate training, surgical expertise, and clinical experience are essential in minimizing postoperative complications. Their removal may result in substantial osseous defects, which often heal slowly and compromise periodontal health and may ultimately lead to the loss of adjacent teeth [5].

As expected, third molar surgery has a pronounced negative impact on patients’ quality of life during the first postoperative week. However, quality of life gradually improves with the resolution of postoperative pain and inflammation [6]. Reducing postoperative complications—particularly pain and swelling following mandibular third molar surgery—has long been a major focus of clinical research [7,8,9,10]. These complications primarily arise from surgical trauma to both hard and soft tissues. Given the incidence and potential severity of such complications following surgical procedures, including third molar extraction, there is a clear need for objective and standardized evaluation of individual therapeutic approaches. To optimize treatment outcomes and improve patient comfort, both clinical and cytomorphometric parameters can be used for postoperative assessment.

In recent years, autologous blood-derived biomaterials have attracted considerable attention due to their regenerative potential in medicine and dentistry [11]. Previous studies have demonstrated their effectiveness in reducing postoperative complications following third molar surgery [12,13]. Platelet-rich fibrin (PRF) is a second-generation platelet concentrate characterized by a simple and cost-effective preparation protocol. The resulting fibrin functions as a combined platelet and immune concentrate, incorporating biologically active blood components that are beneficial in healing and immunity within a single fibrin membrane [14]. PRF has been widely adopted in dental practice [15,16]. This autologous blood biomaterial consists of a dense fibrin network enriched with platelets and leukocytes, along with elevated concentrations of growth factors and cytokines that promote tissue regeneration, particularly in soft tissue healing. PRF has been shown to stimulate angiogenesis, exert anti-inflammatory and osteogenic effects, inhibit osteoclastogenesis, and modulate the inflammatory response of mesenchymal stem cells, including interleukin release [17,18].

The surgical extraction of third molars is an invasive procedure, and postoperative infections occur in 10–15% of cases even with adherence to aseptic protocols. Therefore, antimicrobial therapy remains essential, and the use of penicillin-based antibiotics continues to represent the standard postoperative approach following oral surgical procedures. However, additional prophylactic strategies, including the administration of a single dose of azithromycin, have also been recommended [19,20]. Furthermore, the European Medicines Agency recommends azithromycin for the treatment of periodontal diseases, pericoronitis, acute sinusitis, and related conditions [21]. The immune system regulates bone and bone resorption, thereby acting as a key factor in bone homeostasis. Controlled inflammation and immune responses are essential for bone formation, osseointegration, and successful tissue regeneration. In addition to its antimicrobial activity, azithromycin exhibits significant immunomodulatory effects [20]. Azithromycin demonstrates favorable pharmacokinetic properties, including high tissue penetration and prolonged systemic persistence compared with other antimicrobial agents [21]. Therapeutically relevant concentrations may be maintained for up to 6.5 days, indicating sustained retention in target tissues [22]. Given that postoperative infections most commonly develop within the first few weeks after surgery, the dosing regimen applied in this study is considered appropriate for the prevention of postoperative complications.

Given the aforementioned issues, our aim in this study was to evaluate the efficacy of azithromycin and platelet-rich fibrin (PRF) on inflammatory parameters and wound healing following the surgical extraction of impacted mandibular third molars, compared with standard treatment, using cytomorphometric analyses and clinical parameter assessment. The null hypothesis of the present study was that there would be no significant differences in postoperative inflammation, wound healing, clinical outcomes, or cytomorphometric changes among patients treated with standard therapy alone, platelet-rich fibrin (PRF) adjunctive therapy, or PRF combined with preoperative azithromycin following the surgical extraction of impacted mandibular third molars.

## 2. Materials and Methods

### 2.1. Study Design and Participants

#### Ethics Statement

This randomized clinical trial included healthy patients aged 18–50 treated at the Department of Oral Surgery, Clinic of Dental Medicine, Faculty of Medicine, University of Niš, who required surgical extraction of impacted mandibular third molars. Participation in the study was voluntary. Patients were provided with both verbal and written information regarding the objectives and methodology of the study, as well as the protection of their identity, and they gave written informed consent to participate in the study, which was conducted in accordance with the Declaration of Helsinki and approved by the Ethics Committee of Faculty of Medicine, University of Niš (protocol code: 12-1760-1/2-5), and the Ethical Committee of the Clinic of Dentistry, Medical Faculty, University of Niš (protocol code: 14/1-2023-3 EO). This study was registered retrospectively with the clinical trial registration number ISRCTN36568405 (https://www.isrctn.com/ISRCTN36568405), with the registry name “Evaluation of the effects of azithromycin and platelet-rich fibrin on wound healing after surgical removal of mandibular third molars” (accessed on 29 May 2026).

### 2.2. Determination of Sample Size

The a priori sample size was calculated in G*Power 3.1.9.2 software for a repeated-measures ANOVA within–between interaction (3 groups × 3 time points), assuming a medium effect size (Cohen’s f = 0.25), a two-side α = 0.05, study power of 95%, a correlation of 0.50 among repeated measures, and a nonsphericity correction (ε = 0.50). Under these assumptions, the required total sample size was estimated to be *N* = 87 participants (approximately *n* = 29 per group, rounded to 30 per group). As part of the study protocol, we aimed to recruit 90 participants based on the initial sample size calculation, divided equally into 3 groups. However, a total of 124 participants met the eligibility criteria and were enrolled in the study and analyzed.

### 2.3. Participant Selection

Participant selection was based on clinical examination findings, medical record review, and questionnaire data.

The inclusion criteria were as follows: (1) radiographically confirmed vertically impacted mandibular third molars according to Winter’s classification [23] and the Pell and Gregory classification (Class I, Position B) [24]; (2) healthy patients with no history of systemic or acute infectious diseases; (3) patients with no current medication use or ongoing therapy.

The exclusion criteria were as follows: (1) allergy to penicillin or lidocaine; (2) systemic chronic diseases; (3) presence of cystic lesions, infection in the region of the impacted third molar, odontomas, and syndromes affecting the orofacial region.

**Figure 1:**
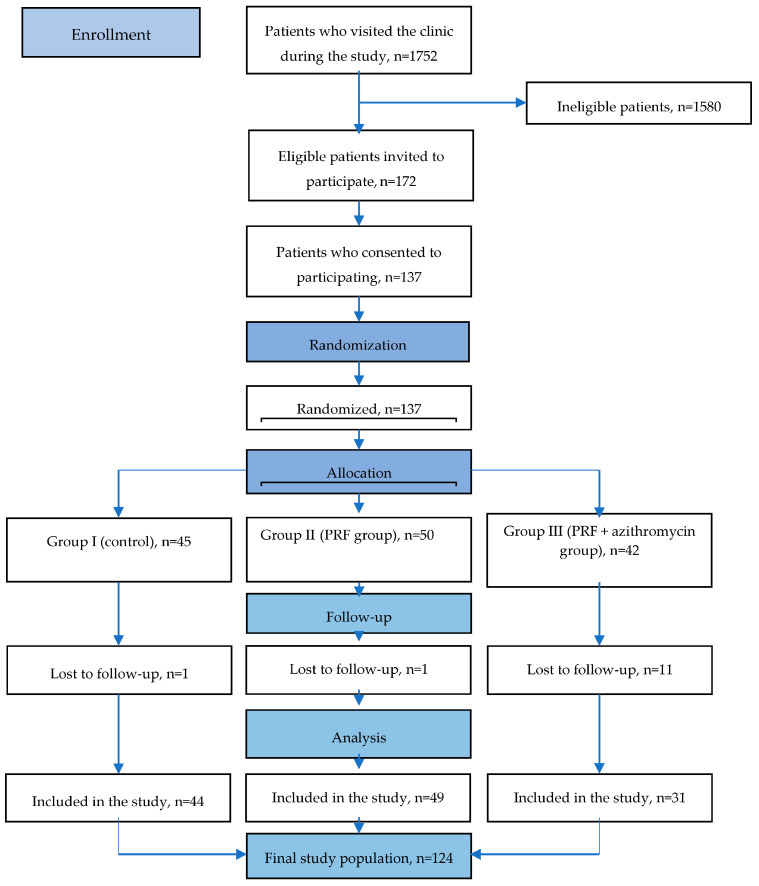
Flowchart of the study.

According to the specified inclusion and exclusion criteria, a total of 172 eligible participants were invited to participate, and 137 patients who consented to participate were randomized; however, the study ultimately included 124 participants (Figure 1).

The participants were divided into three groups:

Group I (control group) included 44 participants with vertically impacted mandibular third molars who received standard postoperative therapy consisting of amoxicillin capsules, 500 mg every 8 h for 5 days, per os.

Group II (examinees) included 49 participants with vertically impacted mandibular third molars in whom postoperative defects were filled with platelet-rich fibrin (PRF), in addition to standard oral therapy with amoxicillin capsules, 500 mg every 8 h for 5 days, per os.

Group III (examinees) included 31 participants with vertically impacted mandibular third molars in whom postoperative defects were filled with PRF, along with standard oral therapy with amoxicillin capsules, 500 mg every 8 h for 5 days, per os, and a single prophylactic dose of azithromycin (1 g) administered one day prior to the procedure.

Randomization was performed using a computer-generated block randomization sequence to promote balanced allocation among the three study groups. Although the randomized groups were similar in size, subsequent losses to follow-up resulted in unequal numbers of participants in the final analysis.

Allocation concealment was achieved using sequentially numbered, opaque, sealed envelopes prepared by an independent examiner who was not involved in participant recruitment, treatment administration, or outcome assessment. Participants and the investigator responsible for recruitment were blinded to group assignment at the time of inclusion. Due to the nature of the intervention, complete blinding of the operator performing the surgical procedure and PRF and azithromycin application was not feasible; this should be considered a limitation of the study. However, all postoperative clinical evaluations were conducted by a blinded outcome assessor who was not involved in treatment delivery. In addition, cytomorphometric analyses were performed by a pathologist using coded samples, and the evaluators were unaware of group allocation. To minimize measurement error and improve reliability, all measurements were repeated twice, and the intraclass correlation coefficient (ICC) was calculated to assess intra-observer agreement.

In addition, all other clinical measurements were performed independently by two trained investigators (doctors of dental medicine). Based on their measurements, inter-observer reliability was evaluated using the interclass correlation coefficient in order to reduce potential assessment and measurement bias.

### 2.4. Data Collection

The study was conducted during the period from April 2024 to September 2025. A detailed medical history was obtained for all participants, followed by clinical and radiographic examinations, and the indication for surgical extraction of impacted mandibular third molars was established. Prior to surgery, all patients underwent basic periodontal therapy and treatment of carious lesions, ensuring that the oral cavity was rehabilitated two weeks before the surgical intervention.

### 2.5. Surgical Procedure

After obtaining informed consent and preoperative preparation, direct local anesthesia was administered via inferior alveolar nerve block and lingual nerve block, with buccal nerve infiltration anesthesia. A 2% lidocaine solution with epinephrine (Galenika, Belgrade, Serbia) was used as the anesthetic agent. The surgical incision was performed using the envelope (sulcular) technique to minimize tissue trauma and preserve the gingival sulcus. An operative incision was made from the distolingual surface of the second mandibular molar horizontally backward along the alveolar ridge for approximately 1.5 cm and along the gingival margin of the adjacent teeth to the level of the second premolar. A No. 15c scalpel blade (SMI, SMI AG, Steinerberg, Belgium) was used for the incision. After elevation of the mucoperiosteal flap, bone removal was performed using a physiodispenser (NSK Surgical, NSK, Tokyo, Japan) and a round carbide bur. The bone overlying the occlusal and buccal surfaces of the impacted mandibular third molar was removed up to the cervical region of the tooth under continuous irrigation and cooling with saline solution. The impacted mandibular third molar was carefully extracted using elevators and forceps without the application of excessive force and without compromising the integrity of the surrounding anatomical structures. The extraction socket was irrigated with 10 mL of sterile saline solution (Galenika, Belgrade, Serbia), and the surgical wound was closed with interrupted 4-0 silk sutures (MSI, Mumbai, India). Following surgery, all study groups received standard postoperative antibiotic therapy consisting of amoxicillin capsules (500 mg every 8 h).

All patients received a standardized postoperative analgesic regimen consisting of a single 500 mg dose of paracetamol administered one hour after surgery. Participants were instructed to use additional analgesics only in cases of severe postoperative pain and to report any such use to the study investigators. No participant reported the use of additional analgesics during the follow-up period. This protocol was applied uniformly across all study groups to minimize variability in postoperative pain management and to ensure comparability across VAS pain assessments.

### 2.6. Venous Blood Collection and Platelet-Rich Fibrin (PRF) Application

In the second group, following debridement, platelet-rich fibrin (PRF) was applied. Venipuncture was performed using standard procedures from the cubital fossa veins (vena mediana cubiti, vena basilica mediana, vena cephalica mediana). A total of 20 mL of blood per patient was collected into two sterile 10 mL tubes without anticoagulant (Pro Cell PRF™, Choukroun, Nice, France) at room temperature (22 °C) [25]. The tubes were immediately centrifuged at 1300 rpm (200× *g*) for 14 min using a fixed-angle rotor centrifuge (Mice8, Colo Lab Experts, Novo Mesto, Slovenia). Following centrifugation, three layers were observed: red blood cells at the bottom, a fibrin clot (PRF) in the middle, and acellular plasma at the top. The PRF clot was retrieved with forceps and separated from the erythrocyte layer using scissors. It was then placed into the defect within a maximum of 15 min. The wound was subsequently closed with interrupted sutures using 4/0 silk suture material (MSI, Mumbai, India).

Patients in the first (control) group were treated using the standard protocol, without PRF application and without a single dose of azithromycin. The surgical wound was closed with interrupted sutures using 4/0 silk suture material (MSI, Mumbai, India).

In the third group, patients received a single prophylactic dose of azithromycin (1 g) the night before surgery, and PRF was placed into the defect [26].

### 2.7. Clinical Assessments

The timing of outcome assessments was predefined according to the clinical characteristics of each parameter. Pain intensity (VAS) was assessed on postoperative days 3 and 7; facial swelling and trismus were evaluated preoperatively (day 1) and on postoperative days 3 and 7; and wound healing was assessed using the Landry Healing Index on postoperative days 3, 7, and 14.

Clinical assessments included the following:

- Pain evaluation using a visual analog scale (VAS) [12,27,28];
- Assessment of facial swelling using standardized facial reference points [12,29];
- Measurement of postoperative trismus by recording the interincisal distance between the incisal edges of the maxillary and mandibular central incisors at maximal mouth opening [12];
- Evaluation of wound healing using the Landry Healing Index [30];
- Cytological and morphometric analysis of gingival tissue in the surgical region, performed immediately before anesthesia and surgery and repeated seven days postoperatively [31].

Pain intensity was recorded by patients using a visual analog scale ranging from 0 (no pain) to 100 (worst imaginable pain) on the third and seventh postoperative days. Postoperative facial swelling was assessed by measuring facial distances between reference points (tragus–gonion and tragus–oral commissure) immediately before surgery (day 1) and on the third and seventh postoperative days in all groups. Postoperative trismus was assessed by measuring the distance between the incisal edges of the maxillary and mandibular central incisors at maximal mouth opening.

The Landry Healing Index was used to assess wound healing parameters, including tissue color in the surgical area, incision margin characteristics (epithelialization), the presence or absence of bleeding on palpation, granulation tissue, and suppuration. Healing levels were assessed as follows: 1, very poor; 2, poor; 3, good; 4, very good; 5, excellent. Evaluations were performed on the third, seventh, and fourteenth postoperative days.

### 2.8. Cytomorphometric Analysis

Cytomorphometric analysis was performed in all participants across the three groups and included cytological and morphometric evaluation of gingival tissue at the surgical site, conducted immediately before surgery and repeated seven days postoperatively [31]. For cytomorphometric analysis, gingival samples from the surgical area were collected using a sterile swab according to standard procedures [32]. The material was smeared onto clean glass slides, evenly distributed in a thin layer, air-dried at room temperature, fixed in 96% ethanol for 10 min, and stained using the Papanicolaou method.

Morphometric analysis was performed using ImageJ software (version 1.53k; National Institutes of Health, Bethesda, MD, USA). Color photomicrographs were acquired using a Nikon DS-Fi1 high-resolution digital camera (Nikon, Tokyo, Japan) attached to a Nikon ECLIPSE 50i microscope (Nikon, Tokyo, Japan). Images were captured at ×40 magnification and converted to 8-bit format prior to analysis. Calibration was performed using a microscopic micrometer scale corresponding to the applied magnification.

For each participant, 100 randomly selected, non-overlapping epithelial cell nuclei were analyzed. The number of microscopic fields examined was not predetermined; rather, as many fields as necessary were evaluated to obtain 100 eligible nuclei per sample. Nuclei with clear boundaries were included in the analysis, whereas overlapping nuclei were excluded. Nuclear contours were manually delineated using a computer mouse, and morphometric parameters were automatically calculated by the software. The mean value of all measured parameters obtained from the 100 analyzed nuclei was calculated for each participant and used for subsequent statistical analysis.

The analyzed variables relating to nuclear shape and size were analyzed, including nuclear area (Area), integrated density (IntDen), aspect ratio (AR), standard deviation of optical density (StdDev), minimum and maximum optical density (Min and Max), nuclear perimeter (Perim), circularity (Circ), Feret’s diameter (FeretX and Feret Y), and nuclear roundness (Roundness).

### 2.9. Statistical Analysis

Data are presented as standard descriptive statistics (mean values, standard deviation, and minimum and maximum values) or as frequency and proportions. Comparisons of numerical variables were conducted using the *t*-test or Mann–Whitney U test, depending on data distribution. Categorical variables were compared using the Chi-square test. Repeated-measures analysis of variance (rm-ANOVA) was used to evaluate changes in outcomes over time across groups. Mauchly’s test was used to evaluate sphericity in rmANOVA. The Greenhouse–Geiser correction was applied when the sphericity was violated. RmANOVA revealed a significant time x group interaction across all evaluated variables except the Landry Index. Therefore, simple effects analyses were conducted to examine within-group and between-group differences at three measurement points. Effect sizes for rmANOVA were reported as partial eta-squared values. Bonferroni correction was used in post hoc pairwise comparisons. The Landry Index was analyzed in within-group comparisons using the Friedman test, followed by a Bonferroni-adjusted Wilcoxon test and between-group comparisons using the Kruskal–Wallis test, followed by the Mann–Whitney test as a post hoc analysis.

Statistical analysis was performed using R software [33]. The interclass correlation was used to assess the measurement reliability of morphometric variables. Except for Feret and MinFeret, which had good reliability (ICC 0.882 and 0.764), all other variables had excellent reliability (ICC > 0.9).

## 3. Results

A total of 124 patients were included in the study (35 males and 89 females). The mean age of the study population was 25.99 ± 8.06 years (range: 18–50 years). The groups were found to be homogeneous with respect to age (*p* = 0.687) and sex (*p* = 0.316) (Table 1).

The values for the examined clinical parameters across the study groups throughout the follow-up period are presented in Table 2.

**Figure 2:**
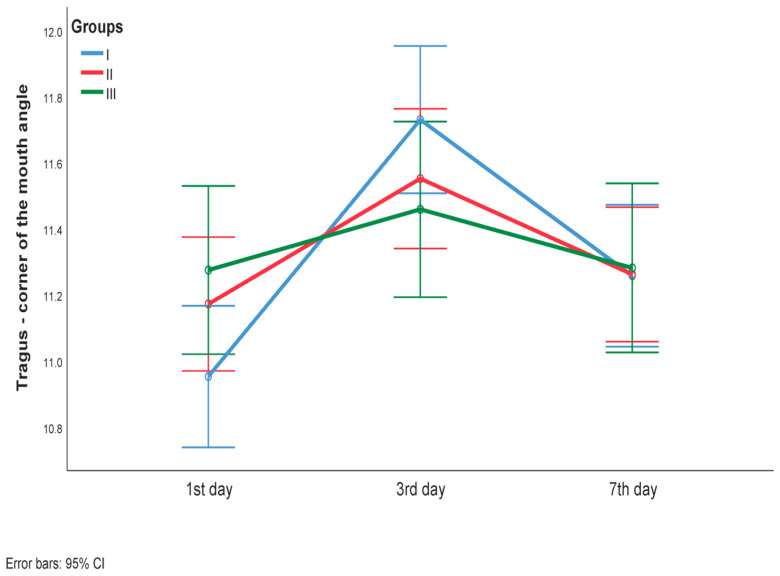
Tragus–corner of the mouth angle during the follow-up period in the study groups.

**Figure 3:**
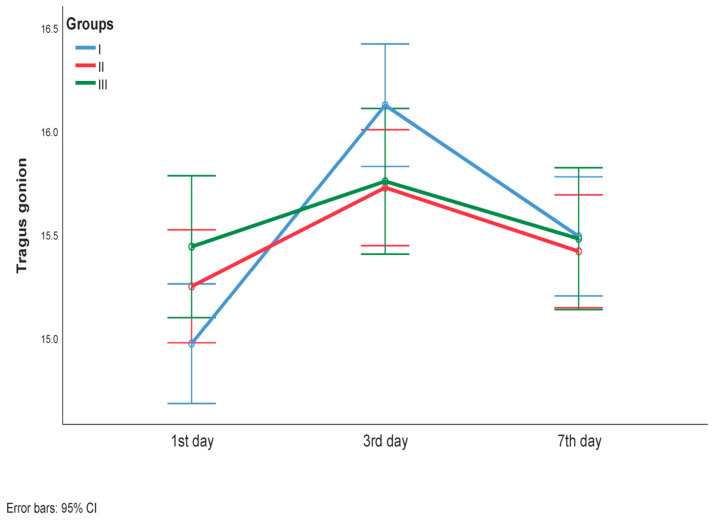
Tragus–gonion angle during the follow-up period in the study groups.

**Figure 4:**
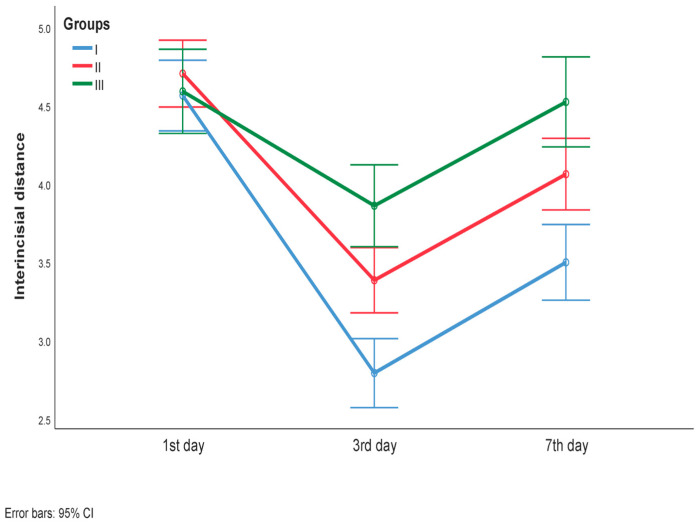
Interincisal distance during the follow-up period in the study groups.

**Figure 5:**
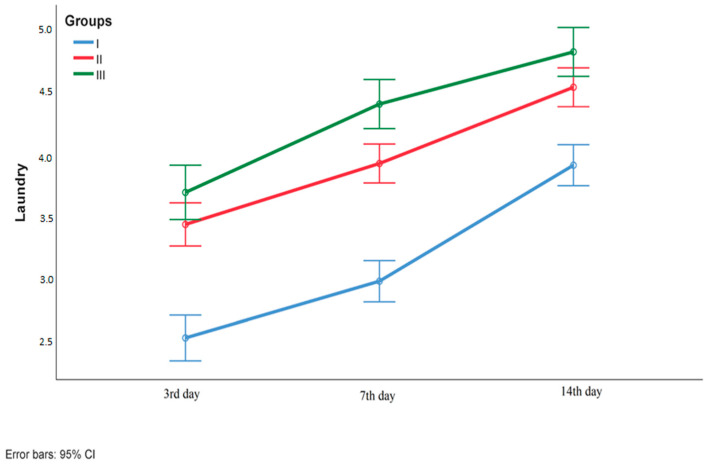
Landry Index values during the follow-up period in the study groups.

Tragus–oral commissure and tragus–gonion distances showed statistically significant changes over the follow-up period (*p* < 0.001), with significant differences observed between all three measurement time points for both parameters (*p* < 0.001 for all) (Figure 2 and Figure 3). The interincisal distance decreased significantly over time (*p* < 0.001), with statistically significant differences between all three measurements (*p* < 0.001 for all) (Figure 4). The Landry Healing Index showed a statistically significant increase over the follow-up period (Friedman test: *p* < 0.001), with significant differences between all three time points (Wilcoxon test: *p* < 0.001 for all) (Figure 5). Additionally, there was a significant increase in this index in each group separately in the follow-up period (Friedman test: *p* < 0.001), with significant differences between all three time points (Wilcoxon test: *p* < 0.001 for all). There were significant differences in the between-group comparisons for each measurement point (Kruskal–Wallis’s test: *p* < 0.001 for all). In addition, Mann–Whitney tests, as a post hoc analysis, revealed that there were significant differences between all groups at each time point (*p* < 0.001 for all), except for the comparisons between Group II and Group III on day 3 (*p* = 0.091) and day 14 (*p* = 0.026). For all measured clinical parameters, a statistically significant difference among the groups and sampling times was observed (*p* < 0.001 for all). Analysis of simple effects demonstrated a statistically significant difference in interincisal distance on day 3 (*p* < 0.001) and day 7 (*p* < 0.001), as well as in Landry Index scores at all three time points (*p* < 0.001 for all).

Post hoc analysis revealed statistically significant intergroup differences in the following parameters: interincisal distance (day 3): I vs II (*p* = 0.001), I vs III (*p* < 0.001), II vs III (*p* = 0.017); interincisal distance (day 7): I vs II (*p* = 0.003), I vs III (*p* < 0.001), II vs III (*p* = 0.043); Landry Index (day 3): I vs II (*p* < 0.001), I vs III (*p* < 0.001), II vs III (*p* < 0.001); Landry Index (day 7): I vs II (*p* < 0.001), I vs III (*p* < 0.001), II vs III (*p* = 0.001); and Landry Index (day 14): I vs II (*p* < 0.001), I vs III (*p* < 0.001).

**Figure 6:**
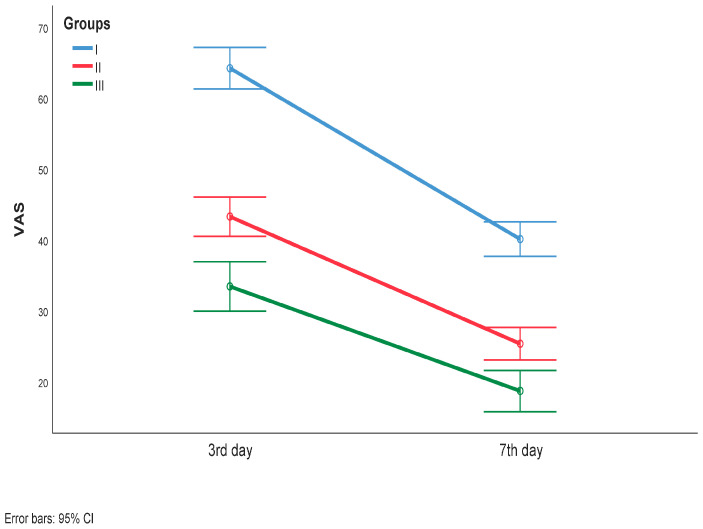
VAS values during the follow-up period in the studied groups.

A statistically significant interaction was observed for VAS scores across the three study groups (rmANOVA, interaction time × group: *p* < 0.001; effect time, *p* < 0.001; and effect group, *p* < 0.001). Analysis of simple effects demonstrated statistically significant differences between all three groups at both measurement time points (*p* < 0.001). Over the follow-up period, VAS scores showed a statistically significant decrease (*p* < 0.001) (Figure 6).

Apart from the MInfaret and Min variables, morphometric parameters did not demonstrate a statistically significant interaction time* × group *p* = 0.038, and *p* = 0.044, respectively. Significant changes over time were observed for the following parameters: AREA (*p* < 0.001), MEAN (*p* < 0.001), Min (*p* < 0.001), Max (*p* < 0.001), Perim (*p* < 0.001), Circ (*p* < 0.001), Feret (*p* = 0.002), RawInden (*p* < 0.001), MinFeret (*p* < 0.001), AR (*p* < 0.001), and Roundness (*p* = 0.001) (Table 3).

## 4. Discussion

The surgical removal of impacted mandibular third molars is frequently associated with complications of varying severity. These range from mild pain and swelling to nerve injury, hemorrhage, severe pain, mandibular fractures, and life-threatening infections [34]. Azena et al. reported complication rates of up to 10.4% [35].

In order to improve wound healing and the regeneration of both soft and hard tissues, autologous blood-derived preparations have been introduced, as they enhance the concentration of growth factors and overall host defense mechanisms.

In the present study, gender distribution was balanced, with minimal and statistically non-significant differences between the groups, and the mean age of the study population was 25.99 years. The mean age of participants in this study is comparable to that reported in similar studies worldwide [36].

The results of this study with regard to healing quality assessed using the Landry Healing Index showed that the control group consistently had the lowest scores at all time points, while the second group demonstrated higher scores with statistical significance, and the highest scores were observed in the third group across all measurements. These findings indicate a significant reduction in inflammatory parameters in the group treated with both azithromycin and PRF, as well as more controlled inflammation in the PRF-only group, which is consistent with previous studies by Alrayyes et al. and Ritto et al. Various forms of PRF have been shown to improve wound closure and healing patterns, as well as reduce postoperative complications through the stimulation of angiogenesis, fibroblast proliferation, and epithelialization [13,30].

Regarding postoperative facial swelling, the most pronounced changes over time were observed in the control group, followed by the second group, which also showed statistically significant increases, whereas no significant increase in facial swelling was observed in the third group. In all groups, facial swelling peaked on the third postoperative day, which is considered a physiological inflammatory response to surgical trauma, as the highest accumulation of inflammatory exudate typically occurs between the second and third postoperative days.

It is well known that PRF contains high concentrations of growth factors that are highly active in promoting angiogenesis and tissue regeneration. When combined with azithromycin, which, in addition to its antimicrobial effects, exhibits immunomodulatory and anti-inflammatory properties, it may further enhance healing and reduce postoperative swelling. This may explain the findings of the present study, in which the third group of patients exhibited minimal and statistically non-significant facial swelling throughout the follow-up period.

With regard to trismus, a statistically significant reduction in mouth opening was observed on the third postoperative day in all groups. The least limitation was observed in the third group, followed by the second and then the control group. This pattern is commonly observed around the third postoperative day, when pain, swelling, and masticatory muscle spasm are most pronounced. During the subsequent postoperative period, trismus gradually resolved in all groups, with the third group showing the values closest to the preoperative measurements.

Overall, the findings of this study regarding facial swelling and trismus are consistent with those reported in previous studies [12].

In a study by Ritto et al., platelet-rich fibrin improved bone density (*p* = 0.007), although no statistically significant difference was found in pain perception assessed using the visual analog scale (VAS). In contrast, the present study demonstrated a significant reduction in pain among patients treated with PRF, particularly in the group receiving both PRF and azithromycin. This discrepancy may be explained by the additional anti-inflammatory and immunomodulatory effects of azithromycin, which likely contributed to a further reduction in inflammation and, consequently, pain [13,37].

Patients in the PRF group (Group II) exhibited lower VAS scores compared to the control group at both measurement points, supporting the analgesic and regenerative potential of PRF. PRF accelerates wound healing by releasing growth factors over an extended period (up to two weeks) and reduces the inflammatory response. Similar findings were reported by Ahmed et al., who demonstrated superior outcomes of PRF in soft tissue healing compared to other treatment modalities [38,39,40]. The lowest pain levels were observed in Group III, suggesting that the combination of PRF and azithromycin exerts an enhanced anti-inflammatory effect, thereby reducing pain. Significant results were also reported by Davido et al., who demonstrated that the combination of azithromycin and PRF exerts a pronounced immunomodulatory effect, improving bone healing and enhancing the success of autogenous bone grafts. This effect is associated with the modulation of the NF-κB signaling pathway [41].

Cytological analyses are highly valuable in assessing inflammatory processes within the oral cavity [42]. The stratified squamous epithelium of the gingiva undergoes continuous desquamation, a process dependent on the mitotic activity of the basal epithelial layer, enzymatic activity within epithelial cells, and mechanical irritation [43].

There is limited literature available on cytomorphometric changes in oral mucosal cells. Existing studies primarily focus on comparisons between smokers and non-smokers, various oral diseases, pharmacological interventions, and prosthetic rehabilitation. Few studies have investigated cytomorphometric changes following third molar surgery. Therefore, the results of the present study contribute to valuable insights into the interpretation of inflammation and soft tissue healing after surgical extraction.

The cytomorphometric findings of this study demonstrated statistically significant changes in the control group at the seventh postoperative day for most of the analyzed parameters (*p* < 0.001). The majority of these parameters reflected changes in cell size, while several also indicated alterations in cell shape. These findings are indicative of an enhanced inflammatory response in the control group, as changes in cellular morphology are known markers of inflammation.

In the analysis of the parameters of the second group, where PRF was placed, no statistically significant changes were observed in any of the analyzed parameters (*p* > 0.05), indicating stability in both cell size and shape over time. This suggests a controlled inflammatory response and more stable tissue healing. In the third group, statistically significant changes were observed in two parameters reflecting dimensional alterations, while the remaining parameters showed no significant differences. These findings suggest that the addition of azithromycin to PRF results in slightly more cytological changes compared to PRF alone but substantially fewer changes compared to the control group.

Area measurements showed an increase in all three groups between preoperative measurements (day 1) and the seventh postoperative day. The smallest change in nuclear size was observed in the third group, which received both azithromycin and PRF, suggesting the presence of a more controlled inflammatory response in this group.

Cytomorphometry is based on the analysis of cellular and nuclear morphology and represents a useful, simple, and non-invasive method for evaluating inflammatory and regenerative tissue changes. In our study, special attention was given to nuclear morphometric parameters, since nuclear size, shape, and chromatin organization reflect cellular metabolic activity and inflammatory responses during healing.

The analyzed parameters (AREA, MEAN, and MinFeret) were used to assess inflammatory and regenerative changes in gingival epithelial cells following the application of PRF and PRF combined with azithromycin. Moderate increases in nuclear AREA may indicate active epithelial regeneration and healing, while excessive enlargement and variability may suggest prolonged inflammation. Similarly, the normalization of MEAN and MinFeret values over time may reflect the stabilization of cellular morphology and a more favorable healing process.

This study has several notable limitations. In addition to the sample size, one of the main limitations is the wide age range of the patients, from 18 to 50 years. Although the majority of participants were between 18 and 35 years of age, a small number of patients were aged 36 to 50 years. Another limitation of the present study is the absence of more objective regenerative outcome measures, such as histological, radiographic, volumetric or three-dimensional imaging techniques, or molecular assessments of tissue healing, since the evaluated parameters were primarily focused on clinical postoperative outcomes and indirect cellular indicators of inflammation and healing. Furthermore, the short follow-up period for wound healing assessment and the absence of monitoring inflammatory mediators represent significant limitations. Therefore, further well-designed studies are needed to overcome these limitations and better define the role of PRF and azithromycin in the wound healing process following the surgical extraction of impacted third molars.

One more important limitation is the absence of an azithromycin-only treatment group. The study design does not allow us to distinguish the independent effects of azithromycin from those associated with its use in combination with PRF. Therefore, any additional benefit observed in the PRF-plus-azithromycin group should be interpreted as a potential synergistic or additive effect rather than as evidence of the isolated efficacy of azithromycin. Future studies including separate PRF-only, azithromycin-only, and combined-treatment groups would provide a more comprehensive evaluation of the individual and combined effects of these therapeutic approaches.

Another important limitation of the present study is that the cytomorphometric findings represent indirect cellular observations and cannot be considered definitive evidence of inflammation control or enhanced tissue regeneration. The study did not include histological analyses, molecular inflammatory markers, radiographic or volumetric assessment of bone healing, or long-term regenerative outcomes. Therefore, the biological significance of the observed cytomorphometric changes should be inferred with caution, and future studies incorporating these objective measures are required to confirm the underlying mechanisms associated with the observed clinical outcomes.

## 5. Conclusions

Based on the results of this study, the greatest trismus and facial swelling were observed on the third postoperative day. Both trismus and facial swelling were least pronounced in the group treated with preoperative azithromycin combined with the intraoperative application of platelet-rich fibrin.

The combination of PRF and preoperative azithromycin was associated with some improvements in postoperative clinical and cytomorphometric parameters compared with the other study groups. However, given the limitations of the present study, including the sample size and follow-up period, these findings should be considered preliminary. Further well-designed studies are required to confirm their potential clinical relevance.

## Figures and Tables

**Table 1:** Demographic characteristics of the study groups.

Groups	Group I		Group II		Group III		*p*
Age	25.84 ± 7.07		25.51 ± 7.42		26.97 ± 8.06		0.687 ^1^
Sex							
Males	9	20.5	15	30.6	11	35.5	0.316 ^2^
Females	35	79.5	34	69.4	20	64.6	

^1^ *t*-test, ^2^ Chi-square test.

**Table 2:** Examined Clinical Parameter Values Across Study Groups During the Follow-Up Period.

Groups	Day 1 #	Day 3	Day 7	*p*		Partial Eta-Squared
Tragus–Corner of the Mouth						
Group I	10.95 ± 0.78	11.73 ± 0.89	11.26 ± 0.82	<0.001	^1^	0.241
Group II	11.17 ± 0.67	11.55 ± 0.61	11.26 ± 0.63	<0.001	^2^	0.529
Group III	11.27 ± 0.70	11.46 ± 0.72	11.28 ± 0.70	0.988	^3^	0.001
Tragus–gonion	Day 1 #	Day 3	Day 7			
Group I	14.97 ± 0.95	16.12 ± 1.10	15.49 ± 0.99	<0.001	^1^	0.357
Group II	15.25 ± 1.01	15.72 ± 0.90	15.42 ± 0.96	<0.001	^2^	0.640
Group III	15.44 ± 0.90	15.75 ± 0.97	15.48 ± 0.93	0.900	^3^	0.002
Interincisal distance	Day 1 #	Day 3	Day 7			
Group I	4.56 ± 0.68	2.79 ± 0.73	3.50 ± 0.78	<0.001	^1^	0.233
Group II	4.70 ± 0.72	3.38 ± 0.70	4.06 ± 0.77	<0.001	^2^	0.700
Group III	4.59 ± 0.91	3.86 ± 0.80	4.52 ± 0.90	<0.001	^3^	0.144
Landry Index	Day 3	Day 7	Day 14			
Group I	2.48 ± 0.76	2.93 ± 0.70	3.86 ± 0.67	<0.001	^1^	0.078
Group II	3.39 ± 0.53	3.88 ± 0.44	4.49 ± 0.51	0.001	^2^	0.719
Group III	3.65 ± 0.49	4.35 ± 0.49	4.77 ± 0.43	<0.001	^3^	0.498
Interincisal distance, 3 days: I vs II, *p* = 0.001; I vs III, *p* < 0.001; II vs III, *p* = 0.017. Interincisal distance, 7 days: I vs II, *p* = 0.003; I vs III, *p* < 0.00; II vs III, *p* = 0.043.						

^1^ interaction time × group; ^2^ time effect; ^3^ group effects; # immediately before surgery.

**Table 3:** Morphometric parameters in repeated measurements between the groups.

Variable	GROUP	M1	M2	*p*		Partial Eta-Squared
**AREA**	1	66.50 ± 13.40	75.42 ± 14.93	<0.001	^1^	0.172
	2	68.06 ± 12.99	83.80 ± 19.29	0.370	^2^	0.025
	3	72.29 ± 17.50	75.96 ± 19.53	0.077	^3^	0.062
**MEAN**	1	0.37 ± 0.06	0.30 ± 0.05	<0.001	^1^	0.337
	2	0.35 ± 0.05	0.32 ± 0.04	0.933	^2^	0.002
	3	0.36 ± 0.04	0.32 ± 0.06	0.112	^3^	0.053
**StdDev**	1	0.04 ± 0.01	0.03 ± 0.01	0.301	^1^	0.033
	2	0.04 ± 0.01	0.04 ± 0.01	0.720	^2^	0.008
	3	0.04 ± 0.01	0.04 ± 0.01	0.055	^3^	0.070
**Min**	1	0.24 ± 0.05	0.19 ± 0.03	<0.001	^1^	0.311
	2	0.23 ± 0.04	0.20 ± 0.04	0.781	^2^	0.006
	3	0.23 ± 0.03	0.20 ± 0.06	0.466	^3^	0.019
**Max**	1	0.45 ± 0.06	0.37 ± 0.06	<0.001	^1^	0.315
	2	0.43 ± 0.06	0.40 ± 0.04	0.868	^2^	0.004
	3	0.43 ± 0.04	0.39 ± 0.07	0.044	^3^	0.075
**Perim**	1	30.30 ± 3.09	31.84 ± 3.01	<0.001	^1^	0.145
	2	30.50 ± 2.96	33.49 ± 3.82	0.437	^2^	0.020
	3	31.23 ± 3.39	31.91 ± 3.76	0.095	^3^	0.057
**Circ**	1	0.90 ± 0.03	0.92 ± 0.03	<0.001	^1^	0.150
	2	0.91 ± 0.02	0.92 ± 0.02	0.260	^2^	0.033
	3	0.91 ± 0.02	0.92 ± 0.02	0.198	^3^	0.040
**Feret**	1	11.28 ± 1.22	11.59 ± 1.06	0.002	^1^	0.071
	2	11.30 ± 1.20	12.17 ± 1.43	0.522	^2^	0.016
	3	11.53 ± 1.17	11.64 ± 1.14	0.139	^3^	0.048
**IntDen**	1	23.97 ± 4.37	22.44 ± 5.86	0.668	^1^	0.001
	2	23.78 ± 5.60	26.15 ± 5.50	0.291	^2^	0.030
	3	25.60 ± 6.45	23.96 ± 6.80	0.072	^3^	0.064
**RawInden**	1	129,013.94 ± 36,129.28	169,034.73 ± 38,763.31	<0.001	^1^	0.330
	2	135,133.11 ± 28,393.09	181,462.41 ± 52,113.51	0.551	^2^	0.015
	3	141,641.95 ± 36,717.95	163,585.25 ± 49,094.12	0.194	^3^	0.040
**FeretX**	1	627.15 ± 128.64	645.19 ± 92.38	0.740	^1^	0.001
	2	632.35 ± 104.69	622.19 ± 91.84	0.766	^2^	0.007
	3	651.37 ± 129.39	631.52 ± 99.63	0.688	^3^	0.009
**FeretY**	1	528.17 ± 100.82	523.78 ± 96.87	0.665	^1^	0.005
	2	506.21 ± 91.88	511.61 ± 85.51	0.466	^2^	0.019
	3	521.59 ± 97.59	488.58 ± 91.84	0.602	^3^	0.013
**FeretAngle**	1	92.00 ± 27.90	83.73 ± 23.35	0.120	^1^	0.020
	2	81.43 ± 28.22	91.16 ± 28.86	0.734	^2^	0.008
	3	75.19 ± 19.63	91.42 ± 33.36	0.114	^3^	0.053
**MinFeret**	1	7.79 ± 0.81	8.63 ± 0.91	<0.001	^1^	0.228
	2	8.00 ± 0.82	9.06 ± 1.12	0.305	^2^	0.029
	3	8.35 ± 1.05	8.54 ± 1.25	0.038	^3^	0.079
**AR**	1	1.42 ± 0.13	1.30 ± 0.12	<0.001	^1^	0.121
	2	1.37 ± 0.14	1.29 ± 0.12	0.287	^2^	0.031
	3	1.34 ± 0.09	1.33 ± 0.11	0.180	^3^	0.042
**Roundness**	1	0.72 ± 0.07	0.78 ± 0.06	0.001	^1^	0.112
	2	0.75 ± 0.07	0.79 ± 0.07	0.311	^2^	0.029
	3	0.76 ± 0.05	0.77 ± 0.06	0.197	^3^	0.040
**Solidity**	1	0.97 ± 0.01	0.97 ± 0.01	0.080	^1^	0.020
	2	0.97 ± 0.01	0.97 ± 0.01	0.317	^2^	0.028
	3	0.97 ± 0.01	0.97 ± 0.01	0.401	^3^	0.023

^1^ time effect, ^2^ group effect, ^3^ time group interaction.

## Data Availability

The original contributions presented in this study are included in the article. Further inquiries can be directed to the corresponding author.

## Abbreviations

The following abbreviations are used in this manuscript:

PRF	platelet-rich fibrin
Area	including nuclear area
IntDen	integrated density
AR	aspect ratio
StdDev	standard deviation of optical density
Min and Max	minimum and maximum optical density
Perim	nuclear perimeter
Circ	circularity
FeretX and Feret Y	Feret’s diameter
Roundness	nuclear roundness
VAS	visual analog scale
